# A Study to Design a Learning Tool “Virtual Patient” for Functional Diagnosis and Clinical Reasoning of Respiratory Dysfunction in the Undergraduate Physiotherapy Curriculum

**DOI:** 10.7759/cureus.35867

**Published:** 2023-03-07

**Authors:** Vaishnavi Yadav, Tripti Srivastava, Waqar M Naqvi, Ankit Bhurane

**Affiliations:** 1 Physiotherapy, Datta Meghe Institute of Medical Sciences, Wardha, IND; 2 Physiology, Datta Meghe Institute of Medical Sciences, Wardha, IND; 3 Community Health Physiotherapy, Ravi Nair Physiotherapy College, Datta Meghe Institute of Medical Sciences, Wardha, IND; 4 Research, NKP Salve Institute of Medical Sciences and Research Center, Nagpur, IND; 5 Electronics and Communication Engineering, Visvesvaraya National Institute of Technology, Nagpur, IND

**Keywords:** oculus quest, simulation in medical education, respiratory dysfunction, functional diagnosis, virtual reality

## Abstract

The aim of the present study was to develop and design software-based “virtual patient” for learning functional diagnosis with clinical reasoning of respiratory dysfunction based on need analysis and perception of faculty and student on utility in the undergraduate physiotherapy curriculum. The objective of the study was to design a framework of a respiratory case scenario that includes personal details, history taking, physical examination, differential diagnosis, investigations, functional impairment, and diagnosis, design a prototype of the virtual patient case scenario using software in a virtual environment created in oculus quest, obtain faculty and student feedback, and analyze the feedback. The result of the study obtained on feedback analysis suggests that the virtual patient case scenario (prototype) contains the relevant information in an organized and sequenced manner. The virtual patient case scenario on the virtual reality platform will be helpful as a teaching and learning modality. The study concluded that the present virtual simulated case scenario (prototype) with more cases helps to develop functional diagnosis and clinical reasoning skills as a part of the undergraduate physiotherapy curriculum.

## Introduction

Artificial intelligence (AI) has progressed sporadically through the years and most recently acquired momentum with the emergence of deep learning and artificial neural networks. The term AI, created by John McCarthy in 1955 [1], is defined as a machine with intelligent behavior such as perception, reasoning, learning, communication, and the ability to perform human tasks [2]. The current applications of AI include its use in automotive, finance and economics, medicine and education including medical education, and Google’s search engine [3,4]. Simulation has been defined as a situation in which a specific type of condition is developed artificially to study or experience something possible in real life or a generic term that refers to the artificial representation of a real-world process to achieve educational goals via experimental learning [5]. The different types of simulations available range from paper-based case scenarios to low- and high-fidelity mannequins. Simulation is the technique that facilitates learning in all domains i.e. cognitive, psychomotor, or affective domains [6]. Simulations are used in several ways including routine learning, rehearsals of communications skills, the practice of complex clinical situations, and introduction into a new clinical environment.

There has been a major change in health profession education worldwide from undergraduate to postgraduate levels [7]. The emphasis is to incorporate all the skills mandatory for the clinical practice including knowledge, skills, and attitude within the framework of a defined curriculum [8]. Both technical and non-technical skills could be assessed and monitored in simulation centers. The traditional methods for teaching clinical and communication skills are difficult to reflect in the clinical postings and do not transfer well to clinical practice [9]. Simulated virtual patients can help in developing all the domains mainly communication skills, consultation skills, physical examination, and diagnosis [10]. The physical signs and symptoms that cannot be simulated can be presented or demonstrated on a simulated virtual patient. The simulated virtual patient can be integrated with high-fidelity (mannequin) simulations or traditional teaching and learning methods to compensate for their limitations [11]. The integration of simulated virtual patients with traditional teaching and learning modality will help in the development of essential skills needed to attain clinical competencies [12]. The hybridization of these two simulations will facilitate analytical thinking, problem-solving skills, diagnostic reasoning, and clinical reasoning [13]. This technique is very useful in teaching many procedural and non-procedural skills to healthcare professionals. Students have the autonomy to plan their learning objectives and learn by reflection.

Revisiting the theories of learning in education is essential while planning for any innovative teaching and learning strategies [14,15]. In health care services around the world, patient safety is of prior importance. Trial and error-based learning cannot be practiced on real patients as far as life is concerned [16]. Recent evidence suggests that medical error represents one of the leading causes of adverse events, mortality, and morbidity [17]. The practice and repetition of any medical procedure or physiotherapy intervention during the learning process are mandatory to reduce error and achieve the desired outcomes [18]. The change in the teaching-learning strategies in recent health profession education by the introduction of innovative methods and too many subjects has made the schedule rigid. This limits the possibilities for students to interact with patients during their clinical postings. This promotes observational learning patterns, due to a lack of opportunities to develop the skill at the patient’s bedside [19]. These situations make it important to bring some changes in the healthcare profession including physiotherapy.

Simulation in the healthcare profession favors the theory of constructivism and recently in practice. It provides the experience of the real world or recreate the original clinical setting and enhance or replace the patient experience [20]. The main advantage of simulation is the freedom to explore and interact under feedback for corrections in an artificial environment. Virtual patients, games, high-fidelity mannequins, and computerized screen-based simulators are available for training purposes that require space, infrastructure, high budget, and standardization [21]. Virtual reality (VR) is emerging as a cost-effective and standardized tool to deliver clinical training. VR is the combination of both hardware and software systems that give the sensory perception of being in the real environment by creating illusions. Recently, VR technologies have been on the verge to bloom into education, teaching, and training in various domains because of their potential for immersion, interaction, and presence [22]. Physiotherapy is the branch that includes comprehensive assessment and evaluation, treatment, and education for movement/functional impairment, bodily malfunction, disability, pain from trauma and disease, healing and physical disorder, and physical and mental conditions using exercise programs, machines, and modalities including exercise, mobilization, manipulations, electro therapeutics for prevention, cure, and rehabilitation [23]. Functional diagnosis and assessment skill is the third-year clinical subject to diagnose movement system dysfunctions and physiological impairments and plan an intervention for health optimization such that the disability can be minimized [24]. Clinical reasoning is the process by which a therapist interacts with a patient, gathers information, creates and tests hypotheses, and gives optimal diagnosis and management based on available data [25].

The study aimed to develop and design a software-based “virtual patient” for learning functional diagnosis with clinical reasoning of respiratory dysfunction based on need analysis. The objective of the study was to assess and analyze the need for virtual patient simulation through personal interviews. The other objective was to design and validate the assessment framework of the respiratory case scenario by subject experts followed by developing a prototype in a VR platform. The final objective was to evaluate the perception of students and faculty members for its utility as a teaching and learning tool through feedback. The prototype designed consists of a virtual patient case scenario with the respiratory disease commonly encountered during clinical postings. The student acted as the physiotherapist examining the virtual patient in a virtual environment using a hand-tracking setting in an oculus quest.

## Materials and methods

Study setting and study design

**Figure 1:**
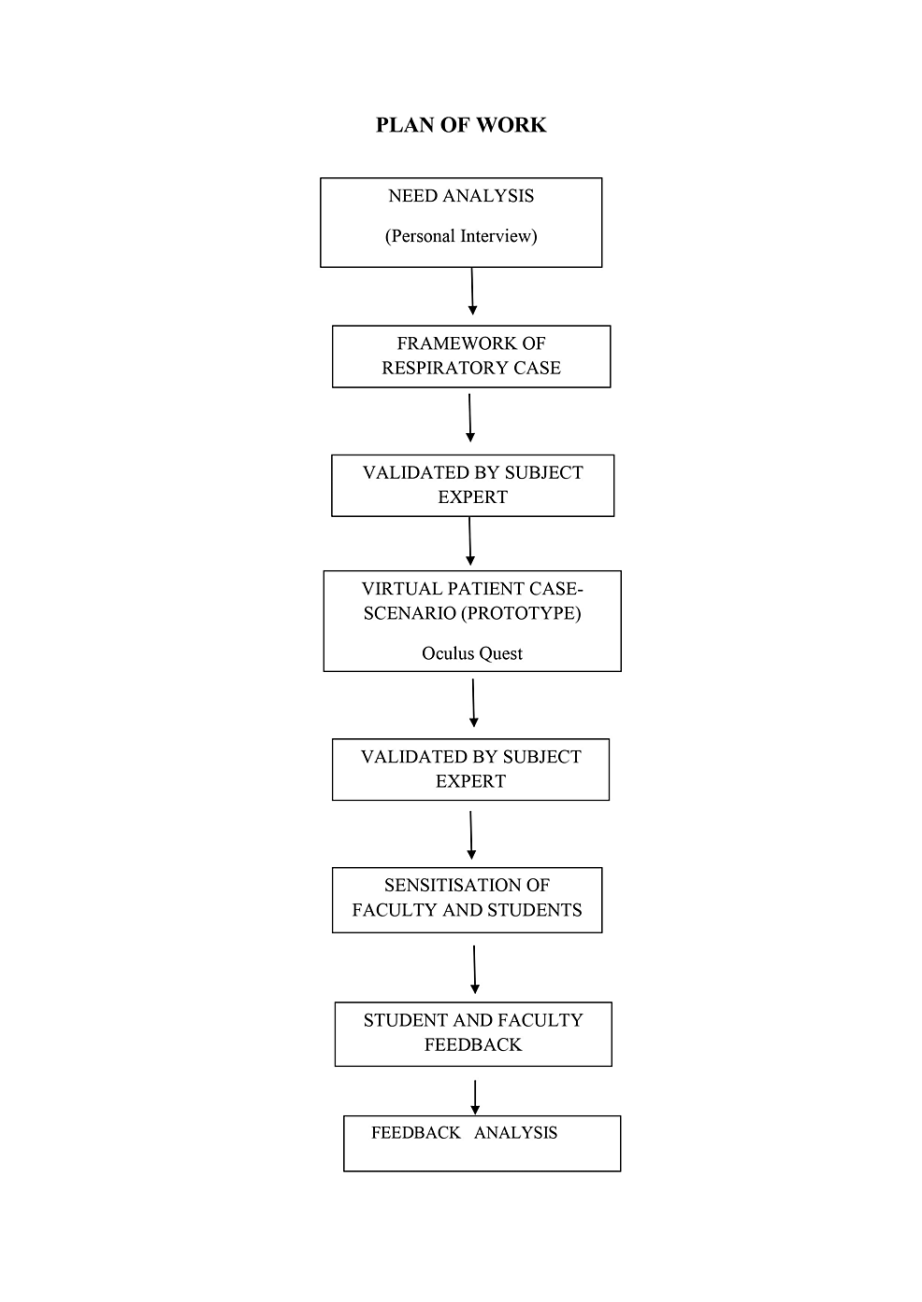
Plan of Work

The study was conducted at Ravi Nair Physiotherapy College, Datta Meghe Institute of Medical Sciences (Deemed to be University) after approval from the Institutional Ethical Committee of Datta Meghe Institute of Medical Sciences (Deemed to be University) with Ethical number- DMIMS (DU)/IEC/May-2019/8787. This is a single-center, prospective observational study of one year that commenced on 1 June 2020. Third and final-year physiotherapy students were included in the study. The personal interviews included 14 subject experts to know the current trends in clinical teaching and the need for any virtual simulation modality. Participants were recruited through convenience sampling including 98 students and 15 faculty members. The prototype of the virtual patient was designed using the validated framework that includes an interview, physical examination, differential diagnosis, and functional diagnosis part. The prepared scenario was again validated by the subject experts. Sensitization of faculty members and the third and final-year physiotherapy students about the simulated virtual patient on oculus quest was done. The questionnaires were prepared to collect the perception of students and faculty members about the virtual patient simulation. The questionnaires were designed with subject experts and experts in the educational unit validated the content. The feedback was obtained and followed by analysis. Fifteen faculty members and 91 students were given the final feedback. The plan of work is shown in Figure 1.

Virtual patient prototype

**Figure 2:**
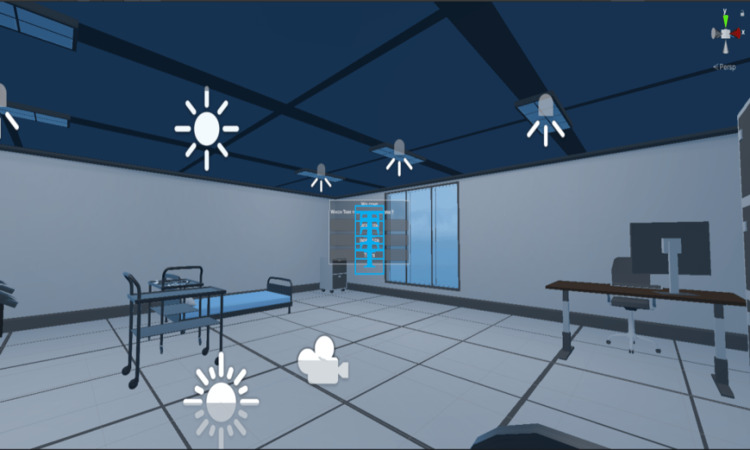
Clinical Virtual Environment

The overall work to develop the simulated virtual patient case scenario includes modeling, rigging, animation, and patient-doctor interaction and grab model interaction, and all are assembled in unity. Modeling was the most basic requirement for this project. In modeling, a model of patients, a clinic room, equipment including a stethoscope, and a thermometer which are required in the hospital wardroom was created (Figure 2).

**Figure 3:**
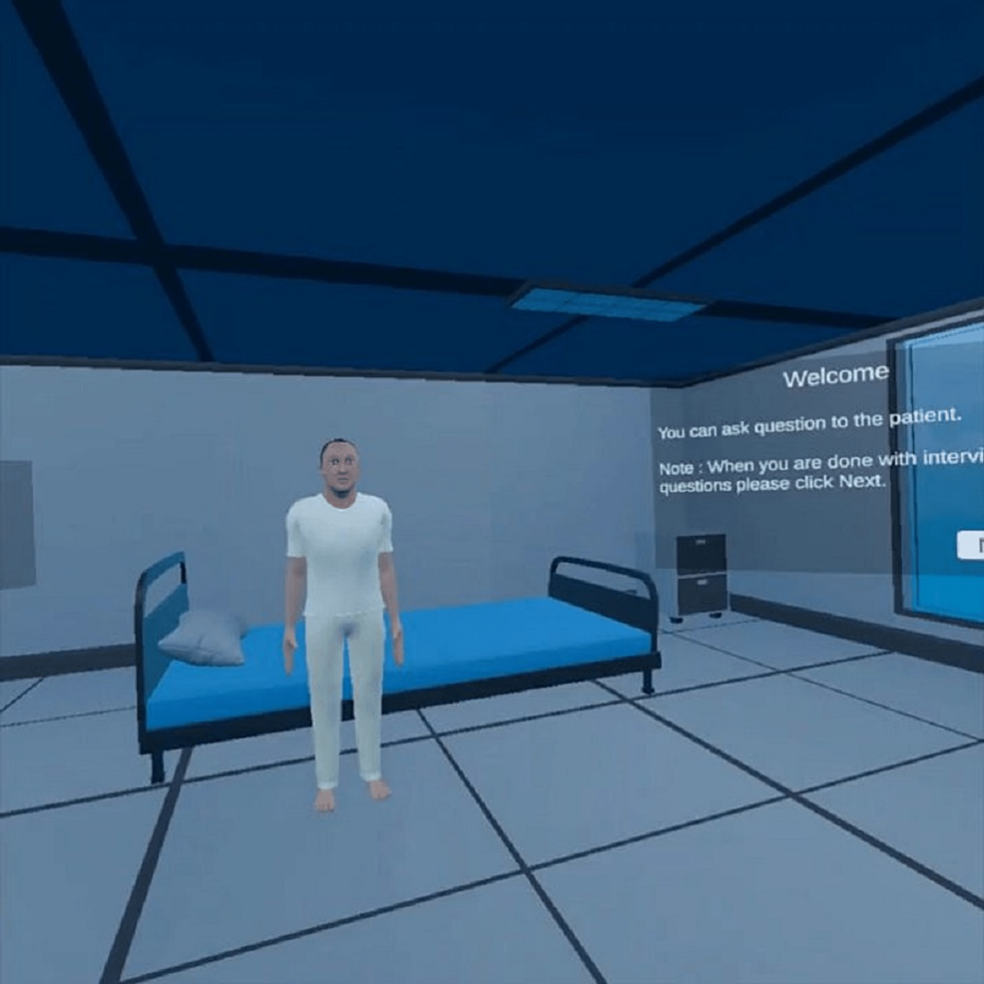
Simulated Virtual Patient

**Figure 4:**
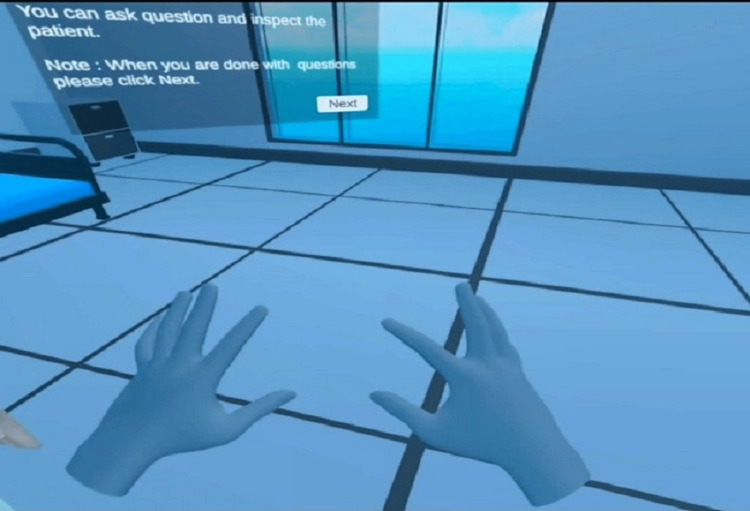
Hand Tracking on Oculus

Rigging of the model is a process very similar to giving bones to a body covered by skin. It is required to have movements in our model as per requirements (Figure 3). This work was done in a software blender as per the requirements of our project. Animation of a model is a process in which the model is trained to move, the animator uses the software as per his expertise. The animation part of this project was to make the patient model standing from a sitting position, sitting back again, walking, breathing, chest movement, etc. This part was developed by the animator and was tested and found to work well. In the doctor-patient interaction part of the project, the aim was to develop an interaction model in which a student wearing an oculus can interact with the patient enabling the student to talk to the virtual patient in a real-time scenario. The student will be asking the question, and the patient will be able to answer at the same time and will be able to perform the task. For the interaction part, around five texts to speech models were tested and desired results were achieved from IBM Watson Model. The interaction model was made compatible with the oculus version. While assessing the patient, the student needs to grab some of the instruments for examination e.g. a stethoscope for auscultation (Figure 4). The model was made compatible to grab using Unity. The different part of the work was assembled on unity software by unity developers. All the models like the clinic room, patient model animations, instrument model, and clinical requirements were combined in unity by developers and made all of them compatible with the VR environment for realism.

## Results

The perception of faculty and students was analyzed using descriptive statistics i.e. percentage. The analysis of the personal interview suggests that the preclinical and clinical students were enthusiastic as well as apprehensive and anxious, and some panicked. The students were not well-developed with the basic skills during their first clinical postings. The students were expected to have some basic clinical skills including history taking, physical examination, and diagnostic and therapeutic skills. They should follow the logical order in an organized manner and conclude with good clinical reasoning skills. Students are expected to be comfortable with the patient and should have good listening skills and empathy toward the patient. The strategies that are currently used to develop these skills are demonstration on models, simulated patients, and practice under supervision. The use of technology that is currently present as e-learning resources includes online videos, websites, Google Scholar, etc. The disadvantages of these are infrastructure and manpower, ethical concerns if directly performed on a patient, communication skills on models and mannequins not possible, standardized patients who do not show any clinical signs and symptoms, and cost of time and training. VR is mainly used in the gaming world and emerging in medical education too. Immersive VR using head-mount displays (HMDs) can give the feel of a real environment that can help students to deal with the real world if designed using the theories of learning and considering the competencies. The virtual patient case scenarios once developed do not need infrastructure and maintenance like high-fidelity mannequins, trial and error are possible, communication skills on models and mannequins are not possible but possible with a virtual patient, and the signs and symptoms specific to disease are possible to be presented in virtual case scenarios. The personal interview of subject experts suggested that immersive virtual reality is a useful tool in limited resource settings and pandemic crises. The results of the student and faculty perception of the virtual case scenario were obtained on a validated questionnaire, shown in Table 1 and Table 2.

**Table 1:** Faculty Feedback for Virtual Patients

Sr.no	Questions	Strongly Agree	Agree	Neutral	Disagree	Strongly Disagree
1.	A virtual patient can run at my own pace and convenience.	-	80%	20%	-	-
2.	A virtual patient is more engaging than the models and mannequins.	-	80%	20%	-	-
3.	Information is in organized and a logical sequence.	-	100%	-	-	-
4.	The virtual patient scenario will facilitate clinical reasoning.	-	100%	-	-	-
5.	Sufficient data are available on history taking part.	-	100%	-	-	-
6.	Interviews with the virtual patient will help in improving the interaction with a real patient.	-	50%	50%	-	-
7.	A physical examination will help in developing psychomotor skills to some extent in a real scenario.	-	50%	50%	-	-
8.	A sufficient number of investigations are available for diagnosis.	-	100%	-	-	-
9.	Sufficient information on medical management is available.	--	100%	-	-	-
10.	Virtual patient facilitates differential diagnosis.		100%	-	-	-
11.	A virtual patient with sufficient relevant information and distracters making little challenging to collect and interpret data.	-	80%	20%	-	-
12.	Virtual patient scenarios can be added to the curriculum as an innovative teaching and learning modality.	-	100%	-	-	-
13.	Virtual patient scenarios can help in developing the competencies to some extent.	-	100%	-	-	-
14.	A virtual patient has scope for error and to learn from mistakes.	-	100%	-	-	-
15.	Virtual patient case scenarios can teach and assess the cognitive domain.	-	100%	-	-	-
16.	Virtual patient meets the desired learning needs and objectives.	-	100%	-	-	-
17.	This kind of virtual patient case scenario will facilitate clinical judgment and clinical decision-making skills.	--	80%	20%	-	-

**Table 2:** Student Feedback

Sr. No.	Questions	Strongly Agree	Agree	Neutral	Disagree	Strongly Disagree.
1.	Virtual patient can create interest and enthusiasm in clinical postings/practice.	46%	43	9%	1%	1%
2.	I can enjoy working on this virtual case scenario.	44%	44%	8%	2%	
3.	Virtual patient will be better for teaching-learning as compared to traditional didactic teaching.	32%	42%	19%	5%	2%
4.	Virtual patient can help in developing analytical thinking.	38%	49%	12%	1%	
5.	Virtual patient can help in facilitating communication skill.	48%	31%	19%	1%	1%
6.	Virtual patient can help in developing analytical thinking.	52%	30%	17%	1%	-
7.	Virtual patient is easy to use.	56%	27%	15%	2%	-
8.	Virtual patient is as realistic as real patient.	51%	23%	24%	2%	1%
9.	Virtual patient scenarios can help in dealing with the same patients in real scenarios to some extent.	24%	61%	15%	-	-
10.	Virtual patient case scenarios can help in understanding the concept easily	64%	26%	9%	1%	-
11.	The Virtual patient scenarios can provide valuable learning experience.	35%	56%	9%	-	-
12.	Virtual patient can help in reducing the anxiety and fear of dealing with patients in reality.	40%	46%	12%	2%	-
13.	Virtual case scenarios should be included in Curriculum as teaching and learning Tool.	34%	57%	9%	-	-

The result of our study obtained by the feedback of faculty and students suggested that a prototype-simulated virtual case scenario can be engaging, interesting, and convenient to use once trained. More simulated cases can help in developing clinical reasoning skills, clinical judgment, and decision-making skills. The present prototype is in an organized and logical sequence, with sufficient data related to history taking part, physical examination, medical management, investigation diagnosis, and functional impairment. Such kinds of case scenarios can be included in the curriculum as innovative teaching and learning modality. The learning needs and objectives can be planned and attained at the end of each case scenario. In the present prototype, the objective was to teach the respiratory assessment, diagnosis, and international classification of functioning to clinical students, also the simulation will be much more useful for preclinical students for early clinical exposure.

## Discussion

The aim of the study was to design a software-based ‘’virtual patient ‘’ for learning functional diagnosis with clinical reasoning in the undergraduate physiotherapy curriculum. The objective of the study was to design the framework for respiratory case scenarios including demographic data, history taking, physical examination, differential diagnosis, investigations, functional impairment, and diagnosis, design a prototype of the virtual patient case scenario using software in a virtual environment created in oculus quest, obtain faculty and student feedback, and analyze the feedback.

Recently, VR has been in trend considering the rehabilitation sector and will be going to bloom in the future. Chavez et al. considered the use of VR in the educational sector for teaching and learning purposes due to its features that allow the implementation of this technology and its useful effect on learning outcomes [26]. The features include a simulated virtual environment, animation, immersion interfaces, interactive capability, and movement. VR in the teaching-learning context has a productive effect on improving learning outcomes, real-time experience, and intrinsic motivation, creates enthusiasm for learning, and enhances skills. The movement feature of virtual reality is very helpful in terms of patient presentation. In the present study prototype, this movement feature has been used for lisping while communicating and breathing as in the case of respiratory conditions.

Jensen et al. reviewed the use of HMDs in education and training for skill acquisition. The authors assessed the various factors affecting immersion, discomfort due to HMDs, learners' attitudes, and barriers to the use of HMDs and stressed the content and areas where HMDs are useful for cognitive, psychomotor, and affective skill acquisition. The authors concluded the content design is poor and more for entertainment purposes and difficult to handle by the teacher. In the present prototype, the content is validated by the subject expert and the framework of the scenario is designed as per the content of the curriculum [27].

As per Botezatu et al., virtual patient simulation is the modality that helps in the achievement of clinical reasoning skills [28]. Janda et al. enlightened the utilization of such instructional methodology in acquiring knowledge and clinical decision-making skill in a controlled environment. This will enhance their confidence during an encounter with a real patient [29].

The creation of virtual patient case scenarios requires robust teamwork; the main is the programmer with the sufficient level of skill to design the environment and code. Furthermore, integration into the curriculum and implementation of such technology requires expert support from both the educational and technical fields [30]. Virtual patient simulation fills a gap between artificial environments toward the real world of clinical care but cannot replace the training on real patients. The main feature of the patient used in the present study is communication in an immersive environment where the student will experience exposure to the real world.

Cost efficiency and cost effectiveness are the major concerns and require collaboration with the incubation centers. Despite these hurdles, the use of training using virtual simulation is growing. Aghili et al. proved that clinical reasoning improves in students working on the application even for a short period. Despite spending a shorter duration on the application, the score was highest in the post-test. This study concluded that compared to the control group, the experimental group working on the application have significant improvement in clinical reasoning [31].

Berman et al. and Huwendiek et al. emphasized that clinical virtual simulation as an adjunct to pedagogical strategy helps to improve clinical reasoning skills by dealing with several case scenarios. The curriculum developer should blend the virtual clinical stimulation with high, low fidelity, or techno simulation as the pedagogical strategies in class. The integration of all forms of simulations to supplement the disadvantages of all can help in the attainment of cognitive, psychomotor, and affective domains of learning [32].

Huwendiek et al. explained the use of clinical simulation in recreating and regenerating the real clinical scenarios in a virtual environment which will help them in a future clinical context. Such modalities enhance the foundation of knowledge and its application in the future. Competency-based education and assessment enable a deep level of learning and development of clinical expertise [33]. Safety and quality of health care can be expected from such simulations by reducing the chances of error [34].

Radianti et al. reviewed work on application of immersive virtual reality in higher education within the context of learning contents, the framework of VR elements, and schools of thought in the learning process as the base for fruitful VR-based learning [35]. The gap analysis found that learning theories are not taken into consideration while designing the framework making it difficult to achieve learning outcomes. The application of VR as a learning tool has mainly focused on its development rather than the outcomes as a teaching-learning tool. The learning content that can be covered includes communication, collaboration and soft skills, procedural and practical knowledge, declarative knowledge, and analytical and problem-solving skill. The study found 18 domains of application indicating huge scope for application in the future. In the present study, we tried to bridge this gap to some extent in the clinical examination of respiratory dysfunction [35].

Gutierrez et al. compared the gain between two groups, one receiving total immersion (head-mounted display) and the other partial immersion (computer screen). The results have proved that VR technology improves knowledge gain significantly in both groups after training experience. However, the fully immersed group showed a significantly greater gain in knowledge than the partial ones. The study concluded that VR has a positive impact on knowledge acquisition, but full immersion is more effective in knowledge structure improvement [36].

Pierce et al. compared the usability of full vs partial immersive virtual reality simulation for medical education and training”. The aim of the study was to determine the most efficient and cost-effective VR simulation technology to achieve the learning objectives. The result of the study found no significant difference in efficiency and level of satisfaction among the fully immersed groups using HMDs as compared to the partially immersed group [37].

The result of our study suggested that a prototype simulated virtual case scenario can be engaging, interesting, and convenient to use once trained. More simulated cases can help in developing clinical reasoning skills, clinical judgment, and decision-making skills. The present prototype is in an organized and logical sequence, with sufficient data related to history taking part, physical examination, medical management, investigation diagnosis, and functional impairment. Such kinds of case scenarios can be included in the curriculum as innovative teaching and learning modality. The learning needs and objectives can be planned and attained at the end of each case scenario. In the present prototype, the objective was to teach the respiratory assessment, diagnosis, and international classification of functioning to clinical students. The simulation will be much more useful for preclinical students as a modality for early clinical exposure.

The limitation of the study

The present study is monocentric, observational and need experimental design further to explore the utility in-depth. A follow-up study with a large sample size including other medical colleges to generalize the results is the future scope.

## Conclusions

Based on feedback analysis, the simulated virtual patient scenario on a VR platform using oculus quest can be used as an adjunct instructional methodology to teach functional diagnosis and clinical reasoning skills in the undergraduate curriculum of physiotherapy students. The case scenarios in the VR platform are organized and structured, which helps in the quick understanding of diseases. Virtual patient scenarios in oculus replace the need for huge infrastructure and fill the gap in currently used simulations. Immersive virtual reality-based case scenarios would be helpful in poor-resource settings and pandemic-like situations. The disadvantage of this tool being not useful in the development of psychomotor and affective domains of learning gives scope for further need in the advancement of technology. A well-designed experimental study is recommended to assess clinical reasoning and learning gain.
